# Family 1 carbohydrate binding-modules enhance saccharification rates

**DOI:** 10.1186/s13568-014-0036-9

**Published:** 2014-04-25

**Authors:** Bruno Luan Mello, Igor Polikarpov

**Affiliations:** 1 Grupo de Biotecnologia Molecular, Instituto de Física de São Carlos, Universidade de São Paulo. Av. Trabalhador Sancarlense, São Carlos CEP 13560-970, SP, Brazil

**Keywords:** Carbohydrate binding-module, Cellulose binding-domain, Enzymatic hydrolysis, Cellulosic ethanol, Amorphogenesis

## Abstract

Cellulose degrading enzymes usually have a two-domain structure consisting of a catalytic domain and a non-catalytic carbohydrate-binding module. Although it is well known the importance of those modules in cell wall degrading process, their function is not yet fully understood. Here, we analyze the cellulose-hydrolysis activity enhancement promoted by the cellobiohydrolase I carbohydrate-binding module from *Trichoderma harzianum*. It was cloned, expressed, purified and used in combination with either a commercial cellulase preparation, *T. reesei* cellobiohydrolase I or its separate catalytic domain to hydrolyze filter paper. In all cases the amount of glucose released was increased, reaching up to 30% gain when the carbohydrate-binding module was added to the reaction. We also show that this effect seems to be mediated by a decrease in the recalcitrance of the cellulosic substrate. This effect was observed both for crystalline cellulose samples which underwent incubation with the CBM *prior* to application of cellulases and for the ones incubated simultaneously. Our studies demonstrate that family 1 carbohydrate-binding modules are able to potentiate the enzymatic degradation of the polysaccharides and their application might contribute to diminishing the currently prohibitive costs of the lignocellulose saccharification process.

## Introduction

Plant cell walls are complex structures, which the main component is cellulose, a linear homopolymer of thousands of glucose residues linked by β-1,4-glycosidic bonds. In plant cell walls, cellulose is found as fibers composed of many chains that pack together by regular H-bonding networks in a crystalline structure, though imperfections and amorphous regions are also observed (Hon [1994]; Knox [2008]). In higher plants, these fibers are encapsulated by hemicellulose and lignin in a structure called lignocellulose (Atalla [1987]).

The enzymatic hydrolysis of cellulose into glucose requires the synergistic action of a set of cellulases traditionally grouped as endoglucanases (EC 3.2.1.4), exoglucanases (EC 3.2.1.91 and 3.2.1.176) and β-glucosidases (EC 3.2.1.21) (Lynd et al. [2002]; Saharay et al. [2010]; Smith et al. [2012]). The glucose released can be then fermented to ethanol by available technologies. Hence, biomass-derived cellulose is a promising feedstock for the production of a second-generation biofuel with little contribution to global warming.

Cellulases from aerobic organisms generally have a two-domain structure consisting of a catalytic domain (CD) and a carbohydrate-binding module (CBM) (Tomme et al. [1988]). CBMs are classified into families based on amino acid sequence similarity and the majority of the fungal cellulases CBMs belong to family 1 ([CAZy]; Cantarel et al. [2009]). It is well known that the CBMs act targeting the CDs to the insoluble substrate and increasing the cellulases local concentration (Reinikainen et al. [1992]; Bolam et al. [1998]; Carrard et al. [2000]). As a consequence, removal of the CBMs reduces dramatically the activity of cellulases toward insoluble substrates even though the activity remains largely unchanged for the soluble ones (Irwin et al. [1993]; Mansfield et al. [1999]; Colussi et al. [2011]). Although in the eighties it was already speculated that CBMs could potentiate polysaccharide deconstruction (Knowles et al. [1987]), the molecular mechanism of such phenomenon is still not clear. While growing body of evidence shows that CBMs from the family 1 might have a disruptive function (Din et al. [1991]; Gao et al. [2001]; Xiao et al. [2001]; Pinto et al. [2004]; Wang et al. [2008]; Hall et al. [2011]) and act to enhance cellulase activity (Gao et al. [2001]; Lemos et al. [2003]; Moser et al. [2008]; Hall et al. [2011]), this effect is frequently not taken into account (Herve et al. [2010]; Varnai et al. [2013]). The elucidation of molecular mode of CBM action is of both scientific and technological importance, since its understanding may lead to a decrease in the cellulase loads used in the paper, textile and bioethanol industries.

Here, we report the use of a family 1 CBM to increase the saccharification of a model cellulosic substrate. To achieve this goal, the CBHI CBM from *T. harzianum* (CBM_CBHI_) was cloned and expressed in *Escherichia coli* fused to a small ubiquitin-like modifier (SUMO). The CBM_CBHI_ was then released from the CBM-SUMO fusion protein by limited proteolysis and purified by size-exclusion chromatography. A commercial cellulase cocktail, the CBHI from *Trichoderma reesei* (TrCBHI) and the CBHI CD from *T. reesei* (CD_CBHI_) were then supplemented with the purified CBM and the activity enhancement on the filter paper hydrolysis was analyzed.

## Material and methods

### Cloning of CBM_CBHI_

Standard molecular biology techniques were used as described elsewhere (Michael and Joseph [2012]). A plasmid cloned previously by our group with the gene for cellobiohydrolase I from *Trichoderma harzianum* [GenBank:AF223252.1] (Bogo et al. [2010]) was used to amplify the CBM_CBHI_ sequence by polymerase chain reaction (PCR) with specific oligonucleotides. The forward primer added LVPRGS thrombin cleavage site to the N-terminal of the CBM_CBHI_ sequence. The forward primer 5′- AAGCTTTACTGGTGCCACGCGGTTCTACACACTACGGCCAG-3′ and reverse primer 5′- CGCGGAACCAGCTCGAGTCATTACAGGCACTGAGAGTAGAATG-3′ contained cut sites for *Hin*dIII and *Xho*I respectively. The PCR product was purified from a 1% agarose gel using the Promega Wizard SV gel purification kit (Promega, Fitchburg, USA). Then, it was cloned into the linear pGEM-T vector (Promega) and transformed into *Escherichia coli* DH5α chemical competent cells. Transformants carrying the CBM_CBHI_ gene were identified by ampicillin resistance and β-galactosidase blue/white screening. pGEM-T + CBM was isolated using Promega Wizard Plus SV miniprep DNA purification system. The recombinant plasmid and pSMT3 expression vector, which encodes a 6His-SUMO N-terminal tag (Mossessova and Lima [2000]), were digested with *Hin*dIII and *Xho*I. The digested fragments were purified from 1% agarose gel as above, ligated using Promega T4 DNA ligase and transformed into *E. coli* DH5α. pSMT3 + CBM was extracted from single colonies that could grow in presence of kanamycin 50 μg/mL, sequenced to be sure there were no unwanted mutations and used to transform *E. coli* Rosetta (DE3) strain. Colonies that could grow in presence of kanamycin 50 μg/mL and chloramphenicol 34 μg/mL were tested for protein production on a small scale. Cells that could overexpress the CBM-SUMO fusion protein were frozen away at -80°C with 20% glycerol.

### CBM_CBHI_ expression and purification

50 mL cultures of *E. coli* carrying the CBM-SUMO gene were grown overnight on 2XYT medium in presence of kanamycin (50 μg/mL) and chloramphenicol (34 μg/mL) at 37°C and used to inoculate 1 L of the same medium. The cultures were grown under constant shaking at 37°C until the optical density at 600 nm reached 0.8. Expression was induced by addition of 1 mM IPTG and carried out for 4 hours. The cells were harvested by spinning down the cultures at 14,000 g for 15 minutes. Next they were suspended in 20 mL of lysis buffer containing 20 mM Tris-HCl, 300 mM NaCl, 5 mM imidazole, 1 mM dithiothreitol (DTT), 1 mM phenylmethylsulfonylfluoride (PMSF), pH 8.0. Finally, the cell suspension was freezed-thawed, sonicated for 6 minutes and centrifuged at 34,000 g for 30 minutes. The supernatant was loaded on a column with 2 mL of nickel-nitrilotriacetic acid (Ni-NTA) resin (Qiagen, Crawley, UK) previously equilibrated with 10 volumes of lysis buffer. The column was washed with 4 volumes of wash buffer (20 mM Tris-HCl, 1 M NaCl, 5 mM imidazole, 5% (v/v) glycerol, pH 8.0) and the recombinant protein was eluted with 4 volumes of elution buffer (20 mM Tris-HCl, 100 mM NaCl, 300 mM imidazole, 5% (v/v) glycerol, 1 mM DTT, pH 8.0). The CBM-SUMO was further purified using Superdex™ 75 16/60 (GE Healthcare Biosciences Corporation, Picataway, USA) column previously equilibrated with 20 mM Tris-HCl, 150 mM NaCl, pH 8.0. The sample purity was determined by sodium dodecyl sulphate-polyacrylamide gel electrophoresis (SDS-PAGE) and Comassie blue staining.

The CBM_CBHI_ was obtained cleaving the fusion protein with 1 U/mg of thrombin at 18°C overnight. It was purified by size-exclusion chromatography as described above.

### Activity assays

The cellulosic substrate Whatman filter paper No. 1 was purchased from Sigma-Aldrich (St. Louis, USA). The TrCBHI, CD_CBHI_ and Accellerase® 1500 (Genencore, Rochester, USA) cellulase preparation were used for hydrolysis. Production and purification of native TrCBHI and its CD_CBHI_ was done following the same protocols used for the homologous protein from *T. harzianum* (Colussi et al. [2010]). Hydrolysis was carried out in PCR plates and 3 mg filter paper discs were used as substrate. To each well 50 μL of CBM-SUMO or CBM_CBHI_ in 50 mM citrate buffer (pH 5.0) was added. The buffer alone and SUMO in 50 mM citrate buffer (pH 5.0) were used as negative controls. The reaction was started by adding 20 μL of Accellerase® 1500, CD_CBHI_ or TrCBHI properly diluted in the same buffer. Reactions were performed in quadruplicate at 50°C. The amount of soluble reducing sugar released by the enzymes was calculated with the dinitrosalicylic acid (DNS) assay (Ghose [1987]) using glucose as a standard. Buffer, protein and substrate blanks were measured for every assay and were subtracted from the experimental data.

## Results

### CBM_CBHI_ expression and purification

**Figure 1:**
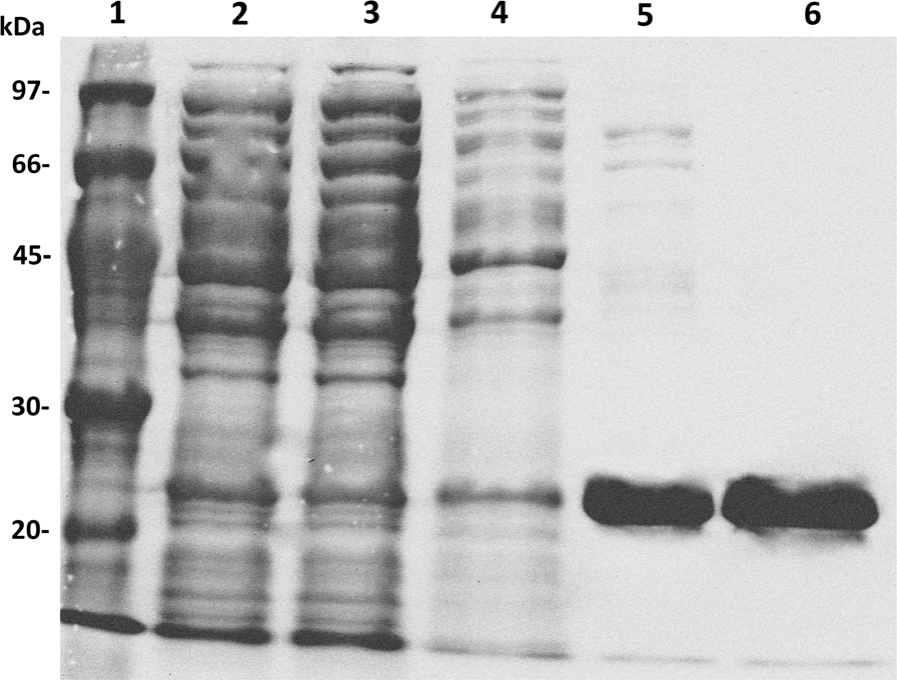
**Expression and purification of CBM-SUMO expressed in*E.coli*Rosetta (DE3).** Lane 1, molecular weight standard; lane 2, lysate soluble fraction from *E. coli* induced with IPTG; lane 3, proteins that did not bind to Ni-NTA resin; lane 4, proteins eluted from Ni-NTA resin with wash buffer; lane 5, protein eluted from Ni-NTA resin with 300 mM imidazole; lane 6, pure CBM-SUMO eluted from size-exclusion chromatography.

The CBM_CBHI_ gene was overexpressed in *E. coli* fused to a 6His-SUMO tag. The tag allowed expression, purification and easier concentration of the protein, as the CBM_CBHI_ has a molecular weight (MW) of only 3.8 kDa and the CBM-SUMO MW is 19.2 kDa. More than 40 mg of the recombinant protein construct was obtained per liter of culture. We also tried to clone and express the CBM_CBHI_ alone, but no expression was detected, probably because small proteins are quickly degraded inside the cell (Cheng and Patel [2004]). Figure 1 shows results of the CBM-SUMO purification steps. After Ni-NTA affinity chromatography the sample was loaded on a gel-filtration column to purify it from high MW contaminants and also from low MW contaminants that were released with the cell lysis but could not be visualized by SDS-PAGE. The fusion protein was then cleaved by thrombin and the released CBM_CBHI_ was purified by gel-filtration chromatography. CBM_CBHI_ elution was monitored by absorbance at 280 nm, but it could not be visualized by SDS-PAGE due to its low MW.

### Hydrolysis reactions

**Figure 2:**
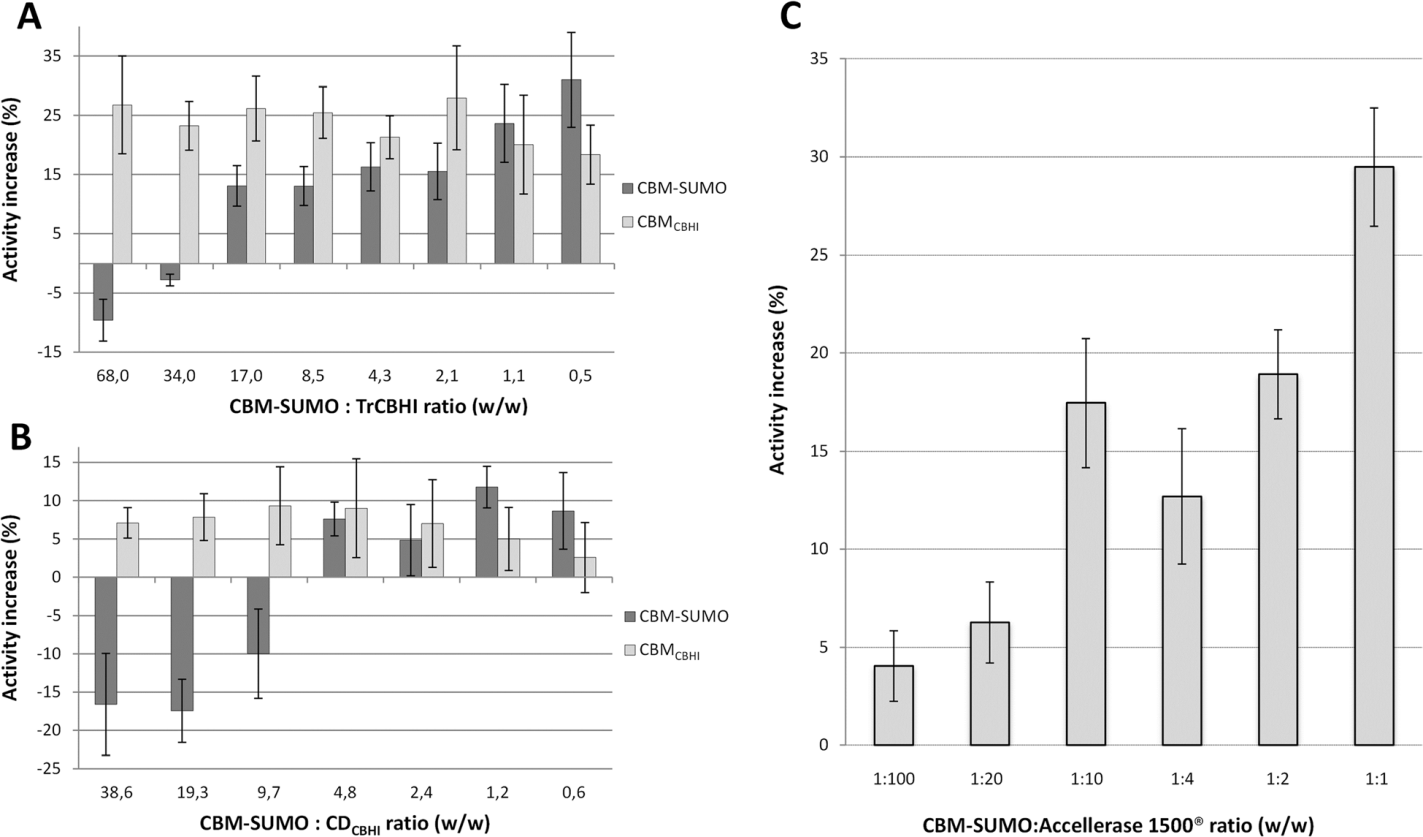
**Effect of increasing amounts of CBM-SUMO or CBM**_**CBHI**_**on filter paper hydrolysis.** The ratios of CBM-SUMO to enzyme varied from 70:1 to 1:100 (w/w). Results from CBM_CBHI_ and CBM-SUMO are grouped based on the molar concentration of those molecules in the reaction. The results are expressed as percentages of the reaction yields in presence of CBM-SUMO or CBM_CBHI_ relative to a negative control experiment carried out without addition of one of these auxiliary proteins. The best enhancement observed was 30%. The cellulase activity after 1 h reaction was around 1 FPU/g of substrate and the enzyme concentration used was **A)** 33.3 μg/mL of TrCBHI, **B)** 58,7 μg/mL of CD_CBHI_ and **C)** 27,5 μg/mL of Accellerase® 1500.

We first incubated different ratios (w/w) of CBM-SUMO or CBM_CBHI_ with TrCBHI or CD_CBHI_ for 1 h and calculated the overall cellulase activity. A clear increase in the rate of filter paper hydrolysis was observed either when TrCBHI was supplemented with CBM_CBHI_ or its SUMO-fusion construct (CBM-SUMO). The boost in TrCBHI cellulase activity was approximately constant and close to 25% (within the experimental errors), no matter what ratios of CBM_CBHI_ to TrCBHI were applied (within 70:1 to 1:2 range of CBM-SUMO:TrCBHI w/w ratios and CBM_CBHI_ at the same molar concentration of CBM-SUMO; Figure 2A). On the contrary, CBM-SUMO effect over TrCBHI activity was negative at very high excess of CBM-SUMO over TrCBHI (-10% at 70:1 w/w ratio, Figure 2A). At smaller CBM-SUMO doses, its addition had positive synergy with the TrCBHI in filter paper hydrolysis, reaching about 30% at 1:2 CBM-SUMO:TrCBHI (w/w) ratio. The TrCBHI activity improvement promoted by CBM-SUMO and CBM_CBHI_ is about the same when the ratio (w/w) of CBM-SUMO:enzyme was 5:1 or less (Figure 2A). Notably, smaller enhancement effect and larger negative contributions at high CBM-SUMO doses were observed when CD_CBHI_ was used in the assays (Figure 2B).

To analyze the influence of the CBM_CBHI_ addition on the activity of commercial enzymatic preparation, we tested its synergy with the Accellerase® 1500. Thus, we incubated different ratios of CBM-SUMO to Accellerase® 1500 for 1 h and calculated the increase in cellulase activity. CBM-SUMO was used in place of CBM_CBHI_ because the previous experiment showed that the cellulase activity improvement of the former construct was the same or higher for ratios (w/w) 5:1 or less of CBM:enzyme. As shown in Figure 2C, there was a steady activity improvement reaching values as high as 30% when a ratio of 1:1 (w/w, CBM-SUMO:enzyme) was used. No reducing sugar could be detected when only CBM-SUMO was incubated with the substrate. We observed an enhancement of about 5% even when the ratio was as small as 1:100. To test a substrate memory effect we compared direct addition of CBM-SUMO to the enzymatic preparation with the pretreatment of the filter paper with CBM-SUMO prior to the enzymatic hydrolysis reaction. We observed the same improvement in both cases. No improvement in the enzymatic preparation activity was detected upon addition of CBM-SUMO when the soluble carboxymethyl cellulose was used as substrate.

**Figure 3:**
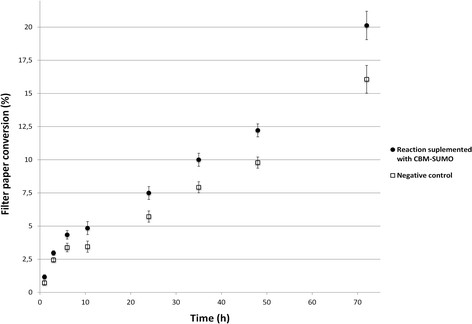
**Hydrolysis profile of filter paper in presence and absence of CBM-SUMO.** The recombinant protein produced an increase in sugar release of about 30% during all the monitored reaction time. Both CBM-SUMO and Accellerase® 1500 concentrations were 27,5 μg/mL.

The best ratio of CBM-SUMO to enzymatic preparation (1:1, on w/w basis) was then used to monitor the hydrolysis of filter paper over time. Surprisingly, the saccharification improvement was about 30% and this gain was constant over time (Figure 3).

## Discussion

Currently, the main function of CBMs is considered to be to target the CDs to the insoluble substrate, thus increasing the effective cellulase concentration on the polysaccharide surface (Reinikainen et al. [1992]; Bolam et al. [1998]; Carrard et al. [2000]). However, there is a growing body of evidence showing that CBMs from bacterial (Din et al. [1991]; Moser et al. [2008]) and fungal cellulases (Gao et al. [2001]; Xiao et al. [2001]; Lemos et al. [2003]; Pinto et al. [2004]; Wang et al. [2008]; Hall et al. [2011]) act in the amorphogenesis of cellulose. On the other hand, several studies reported that CBMs have no disruptive activity (Gill et al. [1999]; Carrard et al. [2000]; Saloheimo et al. [2002]) or do not increase the saccharification rates if incubated simultaneously with a cellulase cocktail (Hall et al. [2011]). To address this contradictory evidence we set out to study the effect of addition of CBMs on the filter paper hydrolysis by the well-studied TrCBHI, its catalytic domain and a commercial cellulase cocktail.

When the ratios (w/w) of CBM_CBHI_ or CBM-SUMO to TrCBHI was 5:1 or less the observed increase in the activity was the same within the experimental errors. This can be explained by the fact that the SUMO does not interact with cellulose, what was confirmed by the negative controls (results not shown). This might be relevant for application point of view because it is easier and cheaper to produce CBM-SUMO than CBM_CBHI_ as the production of the former construct does not involve partial digestion and further purification steps. When the ratio between CBM to TrCBHI was 10:1 or higher the overall cellulase activity decreases for reactions in presence of CBM-SUMO but not for the ones in presence of CBM_CBHI_. As the activity did not decrease when CBM_CBHI_ was added at the same molar concentration, it probably means that the cellulose binding sites were not saturated. However, the SUMO domain of the CBM-SUMO fusion protein is about 4 times bigger than the CBM_CBHI_. Therefore, the CBM-SUMO might lead to a steric hindrance at the cellulose accessible surface before the binding sites saturation was observed. As a consequence, the steric exclusion effect hampered the approximation of TrCBHI to cellulose, thus decreasing the observed activity. This result agrees with the negatively cooperative adsorption of CBHI CBM from *T. reesei* fused to a red-fluorescent protein observed by Sugimoto et al. ([2012]). The effect observed when CD_CBHI_ was used in place of TrCBHI was similar. However, the CBM-SUMO hindrance was stronger and activity–boosting effect was weaker for both CBM-SUMO and CBM_CBHI_ reaching 12% or less.

When CBM-SUMO was incubated with a commercial cellulase preparation, our results clearly show that it does enhance cellulase activity, particularly at low cellulase loadings (about 1 FPU/g of substrate). It is important to mention that although the loading at which considerable enhancement of cellulose hydrolysis was detected is not very high, it is close to or within the range of the loadings currently used for cellulosic biomass deconstruction in cellulosic bioethanol production studies (3 to 15 FPU/g of substrate range) (Taherzadeh and Karimi [2007]). With that in mind, how can we explain that in previous studies no improvement in cellulose hydrolysis was detected when the CBM incubation was simultaneous (Hall et al. [2011])? One possible explanation is that the authors used a higher cellulase loading. Indeed, our experiments clearly show that the CBM-promoted enhancement of cellulosic activities of the commercial enzymatic preparation is dose-dependent. We could not detect significant activity improvements at cellulase loadings of 2 FPU/g of substrate or higher.

What is the molecular basis for such enhancement of cellulase activity? The observed enhancement might be result of hydrogen bond disruption between cellulose chains, rendering the substrate less recalcitrant for hydrolysis as has already been demonstrated for other CBMs (Gao et al. [2001]; Xiao et al. [2001]; Pinto et al. [2004]; Wang et al. [2008]). Our experimental evidence of the boost in enzymatic hydrolysis yields for the crystalline cellulosic substrate which underwent incubation with CBM *prior* to application of cellulases clearly indicate that the synergistic effect between the CBMs and the cellulases should be mediated by the decrease in recalcitrance of the substrate.

Although many more studies are needed to understand in detail the substrate modifications promoted by CBMs, the fact that they can enhance cellulose hydrolysis yields at low cellulase loadings, is clearly very important for development of cost-effective processes of cellulose saccharification, which is one of the major bottlenecks for cellulosic bioethanol production.

Our study demonstrates that incubation of recombinant CBM_CBHI_ (or its fusion construct with SUMO protein) with a cellobiohydrolase, its catalytic domain or a cellulase commercial preparation has a significant impact on cellulose saccharification when the cellulase activity is in the range of 1 FPU/g of substrate. The boost is up to 30% in glucose yield during filter paper hydrolysis. Thus, family 1 CBM addition to cellulase preparations might provide an alternative method to reduce the amount of enzymes needed to deconstruct cellulosic biomass, which might have direct impacts on the paper, textile and bioethanol industries.

## Competing interest

The authors declare that they have no competing interest.
